# Stratified Impacts of the Infodemic During the COVID-19 Pandemic: Cross-sectional Survey in 6 Asian Jurisdictions

**DOI:** 10.2196/31088

**Published:** 2022-03-22

**Authors:** Xi Chen, Fen Lin, Edmund W Cheng

**Affiliations:** 1 The Jockey Club School of Public Health and Primary Care The Chinese University of Hong Kong Shatin Hong Kong; 2 Department of Media and Communication City University of Hong Kong Kowloon Tong Hong Kong; 3 Department of Public Policy City University of Hong Kong Kowloon Tong Hong Kong

**Keywords:** infodemic, information overload, psychological distress, protective behavior, cross-national survey, Asia, COVID-19

## Abstract

**Background:**

Although timely and accurate information during the COVID-19 pandemic is essential for containing the disease and reducing mental distress, an infodemic, which refers to an overabundance of information, may trigger unpleasant emotions and reduce compliance. Prior research has shown the negative consequences of an infodemic during the pandemic; however, we know less about which subpopulations are more exposed to the infodemic and are more vulnerable to the adverse psychological and behavioral effects.

**Objective:**

This study aimed to examine how sociodemographic factors and information-seeking behaviors affect the perceived information overload during the COVID-19 pandemic. We also investigated the effect of perceived information overload on psychological distress and protective behavior and analyzed the socioeconomic differences in the effects.

**Methods:**

The data for this study were obtained from a cross-national survey of residents in 6 jurisdictions in Asia in May 2020. The survey targeted residents aged 18 years or older. A probability-based quota sampling strategy was adopted to ensure that the selected samples matched the population’s geographical and demographic characteristics released by the latest available census in each jurisdiction. The final sample included 10,063 respondents. Information overload about COVID-19 was measured by asking the respondents to what extent they feel overwhelmed by news related to COVID-19. The measure of psychological distress was adapted from the Posttraumatic Stress Disorder Checklist for Diagnostic and Statistical Manual of Mental Disorders 5 (DSM-5). Protective behaviors included personal hygienic behavior and compliance with social distancing measures.

**Results:**

Younger respondents and women (b=0.20, 95% CI 0.14 to 0.26) were more likely to perceive information overload. Participants self-perceived as upper or upper-middle class (b=0.19, 95% CI 0.09 to 0.30) and those with full-time jobs (b=0.11, 95% CI 0.04 to 0.17) tended to perceive higher information overload. Respondents who more frequently sought COVID-19 information from newspapers (b=0.12, 95% CI 0.11 to 0.14), television (b=0.07, 95% CI 0.05 to 0.09), and family and friends (b=0.11, 95% CI 0.09 to 0.14) were more likely to feel overwhelmed. In contrast, obtaining COVID-19 information from online news outlets and social media was not associated with perceived information overload. There was a positive relationship between perceived information overload and psychological distress (b=2.18, 95% CI 2.09 to 2.26). Such an association was stronger among urban residents, full-time employees, and those living in privately owned housing. The effect of perceived information overload on protective behavior was not significant.

**Conclusions:**

Our findings revealed that respondents who were younger, were female, had a higher socioeconomic status (SES), and had vulnerable populations in the household were more likely to feel overwhelmed by COVID-19 information. Perceived information overload tended to increase psychological distress, and people with higher SES were more vulnerable to this adverse psychological consequence. Effective policies and interventions should be promoted to target vulnerable populations who are more susceptible to the occurrence and negative psychological influence of perceived information overload.

## Introduction

### Background

The COVID-19 pandemic has posed unprecedented challenges to public health and daily life worldwide. A cluster of COVID-19 cases was first reported in December 2019 in Wuhan of Hubei Province in China. Due to the proximity and various links to China, COVID-19 badly hit Asia early on. By imposing strict public health measures, some countries in Asia and the Pacific had better performance in containing the spread of COVID-19 compared with the rest of the world. However, the outbreaks of the Delta variant in several Asian countries and regions, including India, Singapore, Taiwan, and Thailand [1], show the difficulty in suppressing COVID-19 even with the implementation of social distancing and the availability of vaccines. As of May 31, 2021, cumulative cases in Asia had reached over 51.07 million—nearly one-third of the total cases reported globally—and the number deaths had reached over 682,000 [2].

The unpredictable course of the pandemic along with prolonged social distancing can negatively affect people’s mental health, regardless of exposure to the disease itself [3]. A study of the general population in Hong Kong between March 2020 and May 2020 revealed that almost two-thirds of the respondents reported depression or anxiety disorders and about one-quarter met the criteria for risk of psychosis [4]. Also, about 45% of South Korean residents experienced moderate or higher symptoms of depression, anxiety, or stress, as shown by a survey from March 2020 to June 2020 [5]. Similarly, a study of the general population in Taiwan in April 2020 found that 55.8% of the participants reported sleep disturbance and 10.8% reported having suicidal thoughts in the previous week [6].

In addition, the psychological impact of COVID-19 has been fueled by an “infodemic,” which refers to “an overabundance of information—some accurate and some not—that makes it hard for people to find trustworthy sources and reliable guidance when they need it” [7]. An infodemic may cause a feeling of information overload when the amount of information to which people are exposed exceeds the optimal level that they can process and understand effectively [8]. Such a feeling of information overload may worsen when the information contains contradictory or uncertain contents [9,10]. As COVID-19 is a sudden disease outbreak with unknown causes and unpredictable course, its related information involves a high level of uncertainty. For instance, the prevention guidance for COVID-19 is ambiguous and changing as experts and authorities present different perspectives, particularly in the early stage of the outbreak [11]. The complexity of COVID-19 information (eg, contains too much scientific jargon) also contributes to a perception of information overload, as processing COVID-19 information requires great cognitive resources.

Although timely and accurate information during the pandemic helps individuals develop adequate risk perceptions, take preventive measures, and reduce mental distress, an infodemic and information overload can trigger unpleasant emotions, cause confusion and distrust among people, and impede effective public health responses [12-14]. Prior research has revealed that health information overload was negatively associated with one’s attitudes and willingness to use medical services. Health information overload may also hinder the dissemination of health knowledge and affect public health decision-making. For example, cancer information overload may increase individuals’ susceptibility to cancer fatalism [14], reduce the willingness to undergo cancer screening [15], and decrease self-management of chronic diseases [16]. Information overload and its related psychological discomfort can lead to information avoidance [17], reduce motivation for information sharing [18], and reduce health behaviors [19]. During the COVID-19 pandemic, people have been exposed to a great deal of information, which not only is based on scientific evidence but also contains misinformation and rumors from unreliable sources. Exposure to too much ambiguous information may pose an obstacle to appropriate responses and undermine trust in health institutions and programs [20]. As such, researchers and public health authorities should be mindful of the causes and consequences of information overload on emergency responses.

To date, only a few studies have investigated the prevalence and consequences of perceived information overload during the COVID-19 pandemic, most of which discussed the effect of information overload on compliance with protective behaviors [21-25]. In light of the psychological and emotional impacts of COVID-19 information (eg, fatigue and anxiety) [26,27], scholars have suggested that “future studies would add more valuable insights if they aim to investigate psychological and emotional responses of information overload” [25]. Although information overload is a stress indicator, previous findings indicated that information overload measured different concepts of perceived stress and the scales for measuring perceived information overload and perceived stress do not overlap [28].

Besides a lack of research on the psychological consequences of information overload about COVID-19, most existing studies only examined the impact of information overload in the general population while ignoring the potentially stratified impact of information overload in different subpopulations. Moreover, the scope of previous studies on information overload is limited. A recent systematic review of health information overload pointed out that most research has been conducted in the United States, focusing on cancer information overload perceived by cancer patients and thus called for extending the scope to other health issues in different contexts [29].

To fill the gaps, this paper aimed to systematically examine the level, associated factors, and psychological and behavioral consequences of perceived information overload about COVID-19 and explore the sociodemographic variances in the susceptibility to and impact of information overload. The data were collected from a large-scale, cross-sectional survey of 10,063 residents in 6 jurisdictions in Asia, including Hong Kong, Taiwan, Japan, South Korea, Singapore, and Thailand, in May 2020. We focused on 4 research questions: (1) Which segments of the population perceived higher levels of information overload during the COVID-19 pandemic? (2) How has information-seeking behavior (ie, the frequency of accessing and perceived trustworthiness of COVID-19 information from different sources) affected the perception of information overload? (3) Would perceived information overload negatively affect psychological well-being and preventative behavior during the pandemic? (4) If so, which subpopulations are more vulnerable to the psychological and behavioral consequences of information overload? The findings would help identify vulnerable groups who are more susceptible to information overload about COVID-19 and its psychological and behavioral consequences and thus contribute to a nuanced understanding of the correlates and consequences of an infodemic and information overload during the COVID-19 pandemic.

### Prior Work

As information overload arises when there is much more information available than an individual’s information processing capacity [30], people who are more attentive to information or have a lower level of cognitive capacity for dealing with relevant information tend to perceive higher levels of information overload. Previous studies have shown that older adults [31] and women [32-35] experienced greater information overload. Also, those who are less educated [31,36,37] and of lower socioeconomic status (SES) [38,39] were more likely to feel overwhelmed by information, which may be due to their lower capacity for processing information.

The empirical results of the relationship between media exposure and information overload were mixed [38,40,41]. Moreover, the effect of various media types on perceived information overload may be different [36]. During the COVID-19 pandemic, information overload was higher among individuals who used social media as a source for COVID-19 information [42,43], perhaps because misinformation and rumors tend to spread more rapidly on social media platforms [44,45]. Thus, we proposed the following hypotheses: Hypothesis 1 (H1) is that the frequency of obtaining COVID-19 information from traditional sources (eg, newspaper, television, and family and friends) is negatively associated with perceived information overload. Hypothesis 2 (H2) is that the frequency of obtaining COVID-19 information from social media is positively associated with perceived information overload.

Although access to timely and quality health information during outbreaks of infectious diseases can effectively contain the spread of diseases and reduce depressive and anxious feelings [46,47], an overabundance of information can make people feel powerless and anxious, experience information fatigue, and use heuristic rather than systematic information processing [17,48]. Prior research has documented the adverse impact of excessive media consumption on psychological well-being during infectious health outbreaks such as the 2014 Ebola outbreak and the swine flu pandemic [49,50]. Likewise, during the current COVID-19 pandemic, people are likely to develop negative emotions when experiencing information overload [51]. Based on the broadly consistent findings of the negative effect of information overload on psychological well-being, we posited the following hypothesis: Hypothesis 3 (H3) is that perceived information overload is positively associated with psychological distress during the COVID-19 pandemic.

Researchers have also investigated the role of various information sources in psychological well-being and coping behavior during the COVID-19 pandemic. Previous studies in mainland China [52] and Taiwan [53] have revealed that exposure to social media was positively associated with mental distress during the COVID-19 pandemic. In contrast, no such relation was found with traditional media (mass and print media) [42]. We thus proposed the following hypotheses about the effect of information sources and psychological well-being during the COVID-19 pandemic: Hypothesis 4 (H4) is that the frequency of obtaining COVID-19 information from traditional sources (eg, newspaper, TV, family, and friends) has a limited effect on psychological well-being during the COVID-19 pandemic. Hypothesis 5 (H5) is that the frequency of obtaining COVID-19 information from social media is positively associated with psychological distress

Despite the well-documented relationship between perceived information overload and psychological distress, limited studies have investigated the socioeconomic differences in the relationship between perceived information overload and psychological well-being. Prior research suggested that confusing and ambiguous information is especially problematic for those experiencing communication inequality, such as the lack of access to relevant health information or the ability to make sense of information [54]. Populations at risk of communication inequality disproportionately consist of older adults, people with low educational levels and incomes, ethnic minorities, and residents of rural areas [54,55]. Thus, structurally vulnerable populations with communication disadvantages may experience higher levels of psychological distress because of perceived information overload. Therefore, we hypothesized the following: Hypothesis 6 (H6) is that the effects of perceived information overload on psychological distress are stronger among older people. Hypothesis 7 (H7) is that the effects of perceived information overload on psychological distress are stronger among respondents with lower SES.

In addition to psychological responses to information overload, people’s health behavior is also likely to be influenced by information overload. Previous studies on health information overload have consistently shown that those who perceive higher information overload are less likely to perform health behaviors [19,56-58]. However, there were mixed findings on behavioral consequences of information overload during the COVID-19 pandemic. A study of 225 participants in Finland showed that perceived information overload had a negative effect on protective health behavior (eg, self-isolation) during the COVID-19 pandemic [59]. Nevertheless, other studies found no direct impact of information overload about COVID-19 and intention to adopt protective behavior [25]. Some studies even found a positive relationship between information overload and the adoption of protective behavior [23,60]. Given the ambiguity in the empirical evidence, we tentatively hypothesized a negative relationship between perceived information overload and compliance behaviors during the COVID-19 pandemic: Hypothesis 8 (H8) is that participants who perceived higher levels of information overload about COVID-19 were less likely to adopt protective behaviors.

## Methods

### Study Design and Data Collection

The data for this study were obtained from a cross-national survey of public attitudes and responses toward COVID-19 in 6 jurisdictions in East and Southeast Asia, including Hong Kong, Taiwan, Japan, South Korea, Singapore, and Thailand. The survey was conducted by a group of scholars at the City University of Hong Kong between May 11, 2020 and May 26, 2020. The 6 regions were selected due to their geographical proximity to mainland China, the original epicenter of the COVID-19 pandemic, and were hardly hit by the pandemic since late January 2020. In late March 2020, they all entered a second wave of the pandemic as more imported cases from Europe and the United States were detected. They are the Tiger economies characterized by relatively high economic development and governing capacity in Asia. Yet, they also vary in regime types, media development, the stringency of public health measures, and effectiveness in containing the pandemic. Thus, these cases provide an ideal mixture of similarities and differences to examine the impacts of information-seeking behavior, perceived information overload, and psychological well-being during the COVID-19 pandemic.

The surveys were completed using online panels provided by a globally recognized professional survey company. The company’s online panels consist of an opt-in list of 56,000 to 1,440,000 individuals relative to the population size in the 6 jurisdictions surveyed in this study. Online panels have been used increasingly among psychological, social, and medical research [61-64]. Using a panel provider for online research can help obtain a representative sample of the required size and facilitate quick completions for time-sensitive projects [65], especially during the COVID-19 pandemic. We reported our methods in line with the Checklist for Reporting Results of Internet E-Surveys (CHERRIES) [66].

For this study, we requested nationally representative samples of around 2000 adults aged 18 years or older in each of the 6 jurisdictions. Age and gender sampling quotas were set to match the latest available census estimates for age and gender in each jurisdiction. Participants were invited through email messages with an embedded link. The panel provider continuously invited participants until the predetermined quota was met. To increase the response rate, participants would get modest monetary rewards upon completion of the survey. Participation was voluntary, and all responses were anonymous. Details of the survey method of this project can be found elsewhere [67].

We developed a questionnaire that includes questions on perceived information overload about COVID-19, information-seeking behavior, psychological well-being, and protective behavior during the pandemic. We conducted a pre-test of the survey questions and modified wordings based on the feedback from the pre-testers. The questionnaire was available in English, Chinese, Korean, Japanese, and Thai for participants from different jurisdictions. A total of 12,062 representative respondents was collected, with approximately 2000 individuals in each jurisdiction. Cases with incomplete information were excluded from the analysis. The final sample size was 10,063 (see Table 1 for more details).

**Table 1:** Sample characteristics.

Variable		Full sample (N=10,063), n (%)	Hong Kong (n=1813), n (%)	Japan (n=1372), n (%)	Singapore (n=1681), n (%)	South Korea (n=1749), n (%)	Taiwan (n=1695), n (%)	Thailand (n=1753), n (%)
**Age (years)**								
	18-29	2444 (24.29)	413 (22.78)	254 (18.51)	400 (23.80)	431 (24.64)	411 (24.25)	535 (30.52)
	30-39	2441 (24.26)	425 (23.44)	304 (22.16)	389 (23.14)	441 (25.21)	408 (24.07)	474 (27.04)
	40-49	2379 (23.64)	470 (25.92)	263 (19.17)	385 (22.90)	451 (25.79)	394 (23.24)	416 (23.73)
	50-59	1970 (19.58)	392 (21.62)	348 (25.36)	346 (20.58)	273 (15.61)	357 (21.06)	254 (14.49)
	≥60	829 (8.24)	113 (6.23)	203 (14.80)	161 (9.58)	153 (8.75)	125 (7.37)	74 (4.22)
**Sex**								
	Male	5258 (52.25)	865 (47.71)	761 (55.47)	867 (51.58)	927 (53.00)	955 (56.34)	883 (50.37)
	Female	4805 (47.75)	948 (52.29)	611 (44.53)	814 (48.42)	822 (47.00)	740 (43.66)	870 (49.63)
**Education**								
	Secondary school or below	1690 (16.79)	518 (28.57)	316 (23.03)	232 (13.80)	169 (9.66)	194 (11.45)	389 (22.19)
	College or above	8373 (83.21)	1295 (71.43)	1056 (76.97)	1449 (86.20)	1580 (90.34)	1501 (88.55)	1364 (77.81)
**Area**								
	Urban	8147 (80.96)	1673 (92.28)	802 (58.45)	1648 (87.33)	1580 (90.34)	1475 (87.02)	1149 (65.54)
	Rural	1916 (19.04)	140 (7.72)	570 (41.55)	213 (12.67)	169 (9.66)	220 (12.98)	604 (34.46)
**Perceived social status**								
	Lower or lower-middle class	4034 (40.09)	947 (52.23)	658 (47.96)	523 (31.11)	867 (49.57)	632 (37.29)	407 (23.22)
	Middle class	4915 (48.84)	628 (34.64)	523 (38.12)	958 (56.99)	719 (41.11)	827 (48.79)	1260 (71.88)
	Upper or upper-middle class	1114 (11.07)	238 (13.13)	191 (13.92)	200 (11.9)	163 (9.32)	236 (13.92)	86 (4.91)
**Employment status**								
	Working full time	6278 (62.39)	1345 (74.19)	725 (52.84)	1155 (68.71)	886 (50.66)	1253 (73.92)	914 (52.14)
	Other	3785 (37.61)	468 (25.81)	647 (47.16)	526 (31.29)	863 (49.34)	442 (26.08)	839 (47.86)
**Housing type**								
	Privately owned housing	6043 (60.05)	1017 (56.09)	893 (65.09)	1210 (71.98)	1152 (65.87)	1200 (70.80)	1310 (74.73)
	Other	4020 (39.95)	796 (43.91)	479 (34.91)	471 (28.02)	597 (34.13)	495 (29.20)	443 (25.27)
**Chronic illness**								
	Yes	1378 (13.69)	198 (10.92)	244 (17.78)	195 (11.60)	343 (19.61)	161 (9.50)	237 (13.52)
	No	8685 (86.31)	1615 (89.08)	1128 (82.22)	1486 (88.40)	1406 (80.39)	1534 (90.50)	1516 (86.48)
**Having pregnant women or older adults (>65 years old) in the household**								
	Yes	2958 (29.39)	535 (29.51)	383 (27.92)	378 (22.49)	377 (21.56)	620 (36.58)	665 (37.93)
	No	7105 (70.61)	1278 (70.49)	989 (72.08)	1303 (77.51)	1372 (78.44)	1075 (63.42)	1088 (62.07)
**Having children aged under 12 years in the household**								
	Yes	2975 (29.56)	533 (29.4)	254 (18.51)	489 (29.09)	376 (21.50)	490 (28.91)	833 (47.52)
	No	7088 (70.44)	1280 (70.6)	1118 (81.49)	1192 (70.91)	1373 (78.50)	1205 (71.09)	920 (52.48)

### Ethical Review

The study was approved by the Human Subject Ethics Committee of the City University of Hong Kong (Ref No: 8-2020-04-E295-18). All necessary participant consent was obtained.

### Measures

Psychological distress was gauged by the Posttraumatic Stress Disorder Checklist for Diagnostic and Statistical Manual of Mental Disorders (DSM-5; PCL-5) [68]. Five items were selected according to their relevance to COVID-19, such as “Having trouble falling or staying asleep” and “Having trouble concentrating.” The responses ranged from 0 = “Not at all” to 6 = “Very much.” The scores of the 5 items were summed to represent psychological distress, with higher values indicating higher levels of psychological distress. The theoretical score range of the variable was 0-30. The internal reliability of the scale was satisfactory (Cronbach α=0.80).

Protective behavior was assessed by asking the respondents to rate on a 7-point Likert scale the level of compliance with 6 protective behaviors suggested by the government (eg, “kept a distance of at least 2 meters to other people” and “wore a mask in public space”) in the past week. Higher scores indicated higher levels of compliance.

Perceived information overload about COVID-19 was measured by the question, “To what extent do you feel overwhelmed by news related to the COVID-19 pandemic?” (0 = “Not at all” to 6 = “Very much”). This item was selected and adapted from the Perceived Information Overload scale [28] and the Cancer Information Overload scale [14,69].

Information-seeking behavior was assessed by the frequency of accessing COVID-19 information from the following platforms: newspapers, television, online news outlets, and social media (jurisdiction-specific examples were included in blanket statements based on the most popular social media tools in each jurisdiction; 0 = “Never” to 6 = “Very frequently”). We also controlled for perceived source credibility and time spent searching for COVID-19 information. Perceived source credibility was gauged by asking the respondents to rate how trustworthy they considered news about COVID-19 from the sources mentioned above on a 7-point Likert scale (1 = “Not at all trustworthy” to 7 = “Very trustworthy”). We also controlled for the average time (in hours) the respondents spent viewing information about COVID-19 each day.

We adjusted for demographic variables, such as age (18-29, 30-39, 40-49, 50-59, ≥60 years) and sex (male vs female). We also included a series of socioeconomic factors, including education (secondary school or below vs college or above), rural or urban residence, employment status (working full-time vs other), and housing type (privately owned housing vs other). We also assessed perceived social status by asking the respondents to declare their perceived social position (lower or lower-middle class, middle class, upper or upper-middle class). In addition, we controlled for the perceived threat of COVID-19 and level of worry about contracting COVID-19. To assess the impact of having a vulnerable household member, we asked whether the respondent lived with a pregnant woman or an adult older than 65 years and whether they had a child aged 12 years or below.

### Statistical Analysis

Descriptive statistics are reported as mean and SD for normally distributed continuous variables or median and IQR in the case of skewed distributions (Table 1). Normality of the distribution of statistics was tested using skewness or kurtosis tests for normality. For the regression analysis, we first used ordinary least squares (OLS) regression to examine the effects of sociodemographic variables and information-seeking behavior on perceived information overload (outcome variable). Next, OLS regressions with robust standard errors were carried out to examine the effects of perceived information overload and information-seeking behavior on 2 outcome variables (psychological distress and protective behavior) after adjusting for various sociodemographic variables. Lastly, 2-way interaction terms were computed between perceived information overload and each sociodemographic factor (ie, age, sex, education, employment, urban or rural residence, perceived social status, and housing types) to examine whether the effects of information overload on psychological distress (outcome variable) varied across different sociodemographic groups. As the main effect of perceived information overload on protective behavior was not significant, we did not further examine the sociodemographic differences in such an effect. We used graphical and numerical tests to verify OLS assumptions, and the results showed no evident violation of OLS assumptions (detailed information is included in Multimedia Appendix 1). To account for potential heteroscedasticity, we obtained robust standard errors in OLS models [70]. All the analyses were performed using Stata 15.0. Unstandardized coefficients with 95% CIs are reported. A *P* value of .05 was set as the level of statistical significance.

## Results

### Sample Characteristics

Table 1 displays the background characteristics of the respondents. In the full sample, about one-half (4885/10,063, 48.54%) of the respondents were under 40 years old. There were slightly more men (5258/10,063, 52.25%) than women. Most respondents had a college education or above (8373/10,063, 83.21%), lived in urban areas (8147/10,063, 80.96%), worked full-time (6278/10,063, 62.39%), and lived in privately owned housing (6043/10,063, 60.05%). About 40% (4034/10,063, 40.09%) of respondents perceived themselves as lower or lower-middle class, while about 49% (4915/10,063, 48.84%) and 11% (1114/10,063, 11.07%) of respondents perceived their status as middle class or upper or upper-middle class, respectively. About 14% (1378/10,063, 13.69%) of respondents had a chronic illness. About one-third (2958/10,063, 29.39%) of respondents had family members vulnerable to COVID-19 (eg, pregnant women, older adults aged over 65 years, children under 12 years old at home).

### Descriptive Statistics

Table 2 presents the description of key variables. Of a theoretical score range of 0 to 35, the mean psychological distress score among the full sample was 14.27 (SD 8.00). Respondents in South Korea reported the highest level of distress (mean 16.21), followed by residents in Hong Kong (mean 14.90), Thailand (mean 14.48), Singapore (mean 13.74), and Japan (mean 13.14). Taiwan residents reported the lowest level of psychological distress (mean 12.83). As for involvement in protective behaviors, the median for the full sample was 6 (out of a range of 1 to 7), with respondents in Taiwan reporting the lowest level of compliance (median 5.55) and respondents in Singapore reporting the highest compliance (median 6.50). The median level of perceived information overload was 3 (0-6; mean 3.35, SD 1.64). Respondents in South Korea (mean 3.96) and Taiwan (mean 2.65) experienced the highest and lowest levels of information overload, respectively. Residents in Japan, Hong Kong, Singapore, and Thailand reported levels of information overload between 3.02 and 3.76. As for media use, respondents were less likely to use newspapers for COVID-19 information (median 3) and more likely to get COVID-19 information from TV (median 5) and online news outlets (median 5), with social media (median 4) and family and friends (median 4) falling in between. As for perceived source credibility, COVID-19 information on traditional media (television, online news outlets, newspapers, and family and friends) was rated by the respondents as more trustworthy (median 5) than that from social media (median 4).

**Table 2:** Descriptive statistics of key covariates (N=10,063).

Key covariates		Results	Range
Psychological distress, mean (SD)		14.27 (8.00)	0-30
Preventative behavior, median (IQR)		6 (6.33-6.67)	1-7
Perceived information overload, median (IQR)		3 (3-5)	0-6
Perceived susceptibility to COVID-19, median (IQR)		5 (4-7)	1-7
Perceived severity of COVID-19, median (IQR)		6 (5-7)	1-7
**Frequency of seeking COVID-19 information from various sources, median (IQR)**			
	Newspaper	3 (1-5)	0-6
	TV	5 (3-6)	0-6
	Online news outlet	5 (4-6)	0-6
	Social media	4 (3-6)	0-6
	Family and friends	4 (3-5)	0-6
**Trustworthiness of COVID-19 information from various sources, median (IQR)**			
	Newspaper	5 (4-6)	1-7
	TV	5 (4-6)	1-7
	Online news outlet	5 (4-6)	1-7
	Social media	4 (4-5)	1-7
	Family and friends	5 (4-6)	1-7

### Sociodemographic Factors and Perceived Information Overload

Table 3 shows the OLS regression models for perceived information overload and sociodemographic variables. The results showed that older respondents were less likely to experience COVID-19 information overload. Compared with those aged 18 years to 29 years, respondents aged between 40 years and 49 years (b=–0.11, 95% CI –0.20 to –0.01; *P*=.02), between 50 years and 59 years (b=–0.13, 95% CI –0.23 to –0.04; *P*<.007), and at least 60 years (b=–0.21, 95% CI –0.33 to –0.08, *P*<.001) expressed lower levels of information overload. Female respondents were more likely to feel information overload (b=0.20, 95% CI 0.14 to 0.26, *P*<.001). In addition, respondents with higher SES were more likely to experience information overload about COVID-19. Compared with those who perceived themselves as lower or lower-middle class, respondents self-perceived as upper or upper-middle class (b=0.19, 95% CI 0.09 to 0.30; *P*<.001) experienced more COVID-19 information overload. Those with full-time jobs also tended to perceive higher information overload (b=0.11, 95% CI 0.04 to 0.17; *P*=.003) than those without full-time work. The relationship between education and perceived information overload was marginally significant, with respondents with at least a college education more likely to be overwhelmed by COVID-19 information. There were no discernible differences in perceived information overload between people living in rural or urban areas and between housing types. In addition, respondents who had pregnant women or older adults (>65 years old) at home (b=0.16, 95% CI 0.09 to 0.23; *P*<.001) and with children under 12 years in the household (b=0.35, 95% CI 0.28 to 0.43; *P*<.001) were more likely to perceive COVID-19 information overload. As for RQ1, about which segments of the population experienced greater information overload, our findings revealed that young people, women, full-time workers, those who perceived themselves as upper or upper-middle class, and those with vulnerable populations in the household were more likely to experience COVID-19 information overload.

**Table 3:** Association between sociodemographic variables and perceived information overload.

Sociodemographic variables			b^a^		95% CI		*P* value
**Age (years)**							
	18-29	Reference		N/A^b^		N/A	
	30-39	0.02		–0.07 to 0.11		.70	
	40-49	–0.11		–0.20 to –0.01		.02	
	50-59	–0.13		–0.23 to –0.04		.008	
	≥60	–0.21		–0.33 to –0.08		.002	
**Sex**							
	Male	Reference		N/A		N/A	
	Female	0.20		0.14 to 0.26		<.001	
**Education**							
	Secondary or below	Reference		N/A		N/A	
	College or above	0.08		–0.01 to 0.17		.08	
**Residential area**							
	Rural	Reference		N/A		N/A	
	Urban	0.05		–0.04 to 0.13		.28	
**Perceived social status**							
	Lower or lower-middle class	Reference		N/A		N/A	
	Middle class	–0.02		–0.08 to 0.05		.67	
	Upper and upper-middle class	0.19		0.09 to 0.30		.001	
**Employment**							
	Other	Reference		N/A		N/A	
	Working full time	0.11		0.04 to 0.17		.003	
**Housing type**							
	Other	Reference		N/A		N/A	
	Privately owned housing	0.01		–0.06 to 0.08		.81	
**Chronic diseases**							
	No	Reference		N/A		N/A	
	Yes	0.18		0.09 to 0.27		<.001	
**Having pregnant women or older adults (>65 years old) in the household**							
	No	Reference		N/A		N/A	
	Yes	0.16		0.09 to 0.23		<.001	
**Having children aged under 12 years in the household**							
	No	Reference		N/A		N/A	
	Yes	0.35		0.28 to 0.43		<.001	
**Survey location**							
	Hong Kong	Reference		N/A		N/A	
	Japan	0.05		–0.06 to 0.17		.37	
	Singapore	0.57		0.47 to 0.68		<.001	
	South Korea	0.94		0.84 to 1.05		<.001	
	Taiwan	–0.41		–0.52 to –0.31		<.001	
	Thailand	0.66		0.55 to 0.77		<.001	
Constant			2.64		2.50 to 2.78		<.001

^a^Unstandardized coefficient.

^b^N/A: not applicable

### Information-Seeking Behavior and Perceived Information Overload

We then examined the effects of using different sources for COVID-19 information on perceived information overload after adjusting for perceived source credibility, average time spent on viewing COVID-19 information, and various sociodemographic variables (Table 4). Inconsistent with H1, the results revealed that seeking COVID-19 information from traditional sources was positively associated with perceived information overload. Specifically, respondents who more frequently sought COVID-19 information from newspapers (b=0.12, 95% CI 0.11 to 0.14; *P*<.001), television (b=0.07, 95% CI 0.05 to 0.09; *P*<.001), and from family and friends (b=0.11, 95% CI 0.09 to 0.14; *P*<.001) were more likely to feel overwhelmed by the information. In contrast, seeking COVID-19 information from online media, including online news outlets (b=0.00, 95% CI –0.03 to 0.03; *P*=.98) and social media (b=0.02, 95% CI –0.00 to 0.04; *P*=.10) was not significant associated with perceived information overload, which showed limited support for H2.

**Table 4:** The association between information seeking behavior and perceived information overload.

Information-seeking behaviors			b^a,b^		95% CI		*P* value
**Frequency of seeking COVID-19 information from various sources**							
	Newspaper	0.12		0.11 to 0.14		<.001	
	TV	0.07		0.05 to 0.09		<.001	
	Online news outlet	0.00		–0.03 to 0.03		.98	
	Social media	0.02		–0.00 to 0.04		.13	
	Family and friends	0.11		0.09 to 0.14		<.001	
**Trustworthiness of COVID-19 information from various sources**							
	Newspaper	–0.00		–0.03 to 0.03		.94	
	TV	–0.05		–0.08 to –0.01		.01	
	Online news outlet	0.05		0.01 to 0.08		.01	
	Social media	0.10		0.07 to 0.13		<.001	
	Family and friends	0.06		0.03 to 0.10		<.001	

^a^The model was adjusted for sociodemographic variables, including age, sex, education, area, perceived social status, employment, housing types, a history of chronic diseases, having pregnant women or older adults (>65 years old) in the household, having children aged under 12 years in the household, and survey locations.

^b^Unstandardized coefficient.

The relationships between perceived source credibility and perceived information overload were mixed. Specifically, people who trusted COVID-19 information on television more experienced lower levels of information overload (b=–0.05, 95% CI –0.08 to –0.01; *P*=.005). In contrast, perceived trustworthiness of COVID-19 information from online news outlets (b=0.05, 95% CI 0.01 to 0.08; *P*=.005), social media (b=0.10, 95% CI 0.07 to 0.13; *P*<.001), and family and friends (b=0.06, 95% CI 0.03 to 0.10; *P*<.001) was positively associated with perceived information overload. The perceived trustworthiness of COVID-19 information on newspapers was not associated with perceived information overload.

### Perceived Information Overload and Psychological Distress

Next, we investigated the effect of perceived information overload and information-seeking behavior on psychological distress. Model 1 in Table 5 shows the OLS regression models for psychological distress and protective behaviors after controlling for sociodemographic variables. As for psychological distress, a positive and significant effect of perceived information overload and psychological distress existed (b=2.18, 95% CI 2.09 to 2.26; *P*<.001), which lent strong support for H3. As for the role of information-seeking behavior in psychological well-being, the results showed mixed support for H4 and H5. Specifically, more consumption of COVID-19 information from newspapers (b=0.58, 95% CI 0.51 to 0.65; *P*<.001) and family and friends (b=0.29, 95% CI 0.18 to 0.40; *P*<.001) resulted in higher levels of psychological distress. In contrast, obtaining COVID-19 information from online news outlets was associated with less distress (b=–0.32, 95% CI –0.44 to –0.21; *P*<.001). The frequency of getting COVID-19 information from TV and social media was not associated with psychological distress. For perceived source credibility, the perceived trustworthiness of information from newspapers (b=–0.27, 95% CI –0.39 to –0.15; *P*<.001) and TV (b=–0.26, 95% CI –0.39 to –0.12; *P*<.001) was negatively associated with psychological distress caused by COVID-19. In contrast, the perceived credibility of information obtained from social media (b=0.56, 95% CI 0.43 to 0.68; *P*<.001) and family and friends (b=0.24, 95% CI 0.11 to 0.37; *P*<.001) was positively related to psychological distress.

**Table 5:** Association between perceived information overload and psychological distress and preventive behaviors.

Variables		Model 1^a^: psychological distress				Model 2^a^: preventive behavior		
		b^b^	95% CI	*P* value	b^b^		95% CI	*P* value
Perceived information overload		2.18	2.09 to 2.26	<.001	0.01		–0.09 to 0.07	.85
**Frequency of seeking COVID-19 information from various sources**								
	Newspaper	0.58	0.51 to 0.65	<.001	–0.06		–0.13 to 0.00	.06
	TV	–0.03	–0.13 to 0.06	.55	0.19		0.10 to 0.29	<.001
	Online news outlet	–0.32	–0.44 to –0.21	<.001	0.37		0.26 to 0.47	<.001
	Social media	0.05	–0.05 to 0.15	.38	0.18		0.09 to 0.28	<.001
	Family and friends	0.29	0.18 to 0.40	<.001	0.01		–0.10 to 0.11	.86
**Trustworthiness of COVID-19 information obtained from various sources**								
	Newspaper	–0.27	–0.39 to –0.15	<.001	0.08		–0.03 to 0.19	.17
	TV	–0.26	–0.39 to –0.12	<.001	0.14		0.01 to 0.27	.03
	Online news outlet	0.05	–0.09 to 0.19	.55	–0.00		–0.13 to 0.13	.98
	Social media	0.56	0.43 to 0.68	<.001	–0.16		–0.27 to –0.03	.01
	Family and friends	0.24	0.11 to 0.37	<.001	0.18		0.06 to 0.31	.004
Perceived susceptibility		0.80	0.71 to 0.88	<.001	0.41		0.33 to 0.49	<.001
Perceived severity		0.30	0.20 to 0.39	<.001	0.55		0.46 to 0.63	<.001

^a^The model was adjusted for sociodemographic variables, including age, sex, education, area, perceived social status, employment, housing types, a history of chronic diseases, having pregnant women or older adults (>65 years old) in the household, having children aged under 12 years in the household, and survey locations.

^b^ Unstandardized coefficient.

### Sociodemographic Differences in the Adverse Psychological Effect of Information Overload

Finally, we assessed whether the impacts of COVID-19 information overload on psychological distress varied by different sociodemographic characteristics. A series of 2-way interaction terms between perceived information overload and sociodemographic variables were computed. Table 6 presents the regression results of psychological distress on interactions between perceived information overload and sociodemographic factors. The interaction between information overload and age groups was not significant, suggesting that the effect of information overload was not dependent on the respondents’ age—such a finding showed limited support for H6. The results revealed a significant sex difference in the psychological consequences of perceived information overload, with women experiencing more psychological distress in the midst of information overload about COVID-19 (b=0.24, 95% CI 0.10 to 0.39; *P*=.001).

As for SES, there was a positive interaction between COVID-19 information overload and an urban residence (b=0.23, 95% CI 0.04 to 0.41; *P*=.02), suggesting that the detrimental effect of COVID-19 information overload on psychological distress was more salient for urban residents than their rural counterparts. The interaction between information overload and middle-class status was also significant (b=0.18, 95% CI –0.02 to 0.33; *P*=.03), suggesting that self-perceived middle-class respondents were more likely to experience psychological distress when faced with COVID-19 information overload than respondents self-perceived as being lower or lower-middle class. Similarly, full-time employees were likely to experience higher levels of psychological distress when perceiving information overload about COVID-19 (b=0.24, 95% CI 0.09 to 0.39; *P*=.002). Also, the association between perceived information overload and psychological distress was more salient among respondents living in privately owned housing than their counterparts residing in other types of accommodation (b=0.20, 95% CI 0.05 to 0.35; *P*=.01). Inconsistent with H7, such results suggested that those with higher SES were more likely to develop psychological distress when experiencing COVID-19 information overload.

**Table 6:** Associations between perceived information overload and psychological distress among Asian populations with different sociodemographic backgrounds.

Models and variables			b^a^		95% CI		*P* value
**Model 1^b^: perceived IO^c^ × age**							
	Perceived IO	2.08		1.93 to 2.24		<.001	
	Age: 18-29 years	Reference		N/A^d^		N/A	
	Age: 30-39 years	–0.67		–1.48 to 0.13		.10	
	Age: 40-49 years	–1.26		–2.07 to –0.46		.002	
	Age: 50-59 years	–1.35		–2.18 to –0.55		.001	
	Age: ≥60 years	–3.23		–4.28 to –2.18		<.001	
	Perceived IO × 30-39 years	0.18		–0.03 to 0.39		.10	
	Perceived IO × 40-49 years	0.18		–0.03 to 0.40		.09	
	Perceived IO × 50-59 years	–0.01		–0.23 to 0.21		.94	
	Perceived IO × ≥60 years	0.13		–0.16 to 0.41		.39	
**Model 2^b^: perceived IO × >sex**							
	Perceived IO	2.07		1.96 to 2.17		<.001	
	Sex: male	Reference		N/A		N/A	
	Sex: female	–1.23		–1.77 to –0.68		<.001	
	Perceived IO × female	0.24		0.10 to 0.39		.001	
**Model 3^b^: perceived IO ×** **education**							
	Perceived IO	2.16		1.98 to 2.34		<.001	
	Education: secondary or below	Reference		N/A		N/A	
	Education: college or above	–0.05		–0.75 to 0.65		.90	
	Perceived IO × college or above	0.02		–0.17 to 0.21		.83	
**Model 4^b^: perceived IO × area**							
	Perceived IO	2.00		1.82 to 2.17		<.001	
	Residential area: rural	Reference		N/A		N/A	
	Residential area: urban	–0.53		–1.22 to 0.16		.13	
	Perceived IO × urban	0.23		0.04 to 0.41		.02	
**Model 5^b^: perceived IO × perceived status**							
	Perceived IO	2.10		1.98 to 2.23		<.001	
	Perceived status: lower or lower-middle class	Reference		N/A		N/A	
	Perceived status: middle class	–0.79		–1.37 to –0.20		.008	
	Perceived status: upper and upper-middle class	0.62		–0.27 to 1.52		.17	
	Perceived IO × middle class	0.18		0.02 to 0.33		.03	
	Perceived IO × upper or upper-middle class	–0.07		–0.31 to 0.16		.53	
**Model 6^b^: perceived IO × employment status**							
	Perceived IO	2.03		1.91 to 2.16		<.001	
	Employment status: non-full time work	Reference		N/A		N/A	
	Employment status: working full time	–0.76		–1.33 to –0.20		.008	
	Perceived IO × working fulltime	0.24		0.09 to 0.39		.002	
**Model 7^b^: perceived IO × housing types**							
	Perceived IO	2.06		1.93 to 2.18		<.001	
	Housing type: non-privately owned housing	Reference		N/A		N/A	
	Housing type: privately owned housing	–0.77		–1.34 to –0.21		.007	
	Perceived IO × privately-owned housing	0.20		0.05 to 0.35		.01	

^a^Unstandardized coefficient.

^b^All the models adjusted for sociodemographic variables, including age, sex, education, area, perceived social status, employment, housing types, a history of chronic diseases, having pregnant women or older adults (>65 years old) in the household, having children aged under 12 years in the household, and survey locations. The model also controlled for perceived susceptibility to and perceived severity of COVID-19.

^c^IO: information overload.

^d^N/A: not applicable.

### Perceived Information Overload and Protective Behavior

Model 2 in Table 5 shows that the association between perceived information overload and adoption of protective behaviors was not significant after adjusting for sociodemographic variables and perceived susceptibility and severity of the pandemic (b=0.00, 95% CI –0.01 to 0.01; *P*=.88). Such results did not support H8. More frequent exposure to COVID-19 information on TV (b=0.33, 95% CI 0.01 to 0.04; *P*<.001), online news outlets (b=0.06, 95% CI 0.04 to 0.08; *P*<.001), and social media (b=0.03, 95% CI 0.01 to 0.04; *P*=.001) could promote the adoption of protective behaviors against COVID-19. Also, respondents who perceived higher trustworthiness of COVID-19 information obtained from TV (b=0.03, 95% CI 0.01 to 0.05; *P*=.007) and family and friends (b=0.03, 95% CI 0.01 to 0.05; *P*=.008) were more likely to take protective measures. In contrast, there was a negative association between perceived trustworthiness of COVID-19 information on social media and engagement in various protective behaviors (b=–0.02, 95% CI –0.04 to 0.00; *P*=.04).

## Discussion

### Principal Findings

This study is among the first to investigate the antecedents and consequences of information overload about COVID-19 among Asian populations. Using data from a cross-sectional survey of 10,063 residents of 6 jurisdictions in East and Southeast Asia, our study showed a high level of perceived information overload during the pandemic. Regression results further revealed that young people, women, people with a higher SES (ie, full-time workers, self-perception as being upper or upper-middle class), and those with vulnerable populations in the household were more likely to experience COVID-19 information overload. As for the behavioral consequence of information overload, the results showed no significant relationship between perceived information overload and protective behaviors during the pandemic. Consistent with previous studies, we found a positive relationship between perceived information overload and psychological distress. Notably, the association between perceived information overload and psychological distress was more substantial among women and people with a higher SES (urban residents, self-perceived as middle class, full-time workers, and people living in privately owned housing). The findings of this study contribute to a better understanding of the level and correlates of information overload during the COVID-19 pandemic and help identify subpopulations that are particularly susceptible to information overload and its potential downstream consequences. We discuss the main findings in the following sections.

### Comparison With Prior Work

Although the occurrence of information overload has been documented in other disease outbreaks, the level and consequences of information overload during the current global pandemic are unparalleled. On the one hand, the unprecedented scale and impacts of COVID-19 on public health and individual lives have led to intensive media coverage. Other epidemics such as Zika, Ebola, Middle East Respiratory Syndrome (MERS), and H1N1 (swine flu) have caused great damage in daily life. Still, the level of panic caused by COVID-19 is much more severe and has resulted in a large volume of news attention to COVID-19. Also, the high scientific uncertainty and rapidly evolving settings of COVID-19 create opportunities for generation of ambiguous, inaccurate, and conflicting information, which may amplify information overload. On the other hand, the media environment and spreading of information have significantly changed over the past several decades. When another deadly viral disease—Severe Acute Respiratory Syndrome (SARS)—broke out in 2003, none of the major social media outlets was present. In pandemics before the social media era, a multilayered process involving careful review by experts, editing by journals, and releasing essential information under embargo to journalists who seek third-party comments would be performed before releasing scientific knowledge to the public [71]. In contrast, social media platforms can host an enormous amount of user-generated content. During the COVID-19 pandemic, there has been a rush to publish unverified information or even deliberately spread misinformation on various social media platforms, which is likely to cause panic and noncompliance to preventive behaviors.

As for the first research question about which segments of the population perceived higher levels of information overload during the COVID-19 pandemic, our results showed that younger people (18-39 years old), women, and respondents with higher SES (having a college education or above, having a full-time job, and self-perceived as upper or upper-middle class) expressed higher levels of perceived information overload about COVID-19. The finding that women tended to experience more information overload about COVID-19 than men is consistent with findings of previous studies [40-42]. Globally, 70% of the workers in the health and social sectors are women [72]. Women provide the majority of health services and are more likely to take the caregiving lead in the family. Women’s caregiving roles and higher risk exposure during the pandemic have prompted them to pay more attention to COVID-19 information and take COVID-19 more seriously than men [73,74].

Though not consistent with previous studies [31], the finding that older people experienced lower levels of information overload about COVID-19 may be justified by the following reasons. First, increased age is often associated with decreased motivation for health-related information seeking, especially from online media, as older adults typically have lower levels of internet literacy and may experience more difficulties navigating websites [75]. Moreover, older people may be more experienced with information content, even on online platforms. In contrast to younger online users, who mainly browse entertaining and social network sites, older adults are more likely to use the internet for information purposes [76]. Thus, the older generation is less likely to feel cognitively overloaded as they may not seek COVID-19 information as frequently as young people, and they are more experienced in dealing with information [45].

Although several studies have suggested that people with higher SES may experience less information overload [31,36-39], our results showed a positive relationship between education, employment, and perceived social status with perceived information overload about COVID-19. Such unexpected findings may be due to the widening digital divide between socioeconomic groups during the COVID-19 pandemic. The divides in internet coverage and quality of service have exposed people to different levels of life disruptions caused by the pandemic. Although those with adequate internet infrastructure and capacity can work from home, engage in online teaching and learning, and order food and groceries online, people with limited digital access and skills can hardly overcome the economic hardship and life inconvenience caused by the COVID-19 pandemic. More importantly, the costs for timely information for digital “have-nots” tend to be high, as they may need to wait for information to arrive informally rather than obtaining the information online in real time. Given that people with lower SES are more likely to be digitally disadvantaged, they may be involved in fewer information-seeking behaviors and, consequently, less likely to feel overwhelmed by COVID-19 information.

Moreover, the socioeconomic disparities in perceived information overload may gradually emerge as the pandemic unfolds. A study of South Korean residents found no sociodemographic differences in perceived information overload about COVID-19 during the early stage of the pandemic [25]. It may be because their study was conducted at the peak of media coverage of COVID-19 in South Korea (mid-March 2020), and there may be a ceiling effect of perceived information overload when most people were highly attentive to the COVID-19 information. Moreover, the data collection was confined to Seoul residents, and the sample overrepresented young and highly educated people, which limited the socioeconomic differences in the perception of information overload. In contrast, our study was carried out in May 2020 when the 6 jurisdictions surveyed were in their fifth month of the pandemic. Our findings suggested that the disparities in the perception of information overload may gradually emerge as the pandemic unfolds. Also, our study included representative samples in the 6 jurisdictions in East and Southeast Asia, which reduced sampling bias and increased the generalizability of the findings to the target population. Future studies may further examine whether the current results are consistent across different contexts and stages of the pandemic.

In addition, respondents with vulnerable family members were more sensitive to the threats from COVID-19 and, accordingly, were more attentive to relevant information. Our results support that argument by revealing that respondents with vulnerable significant others at home (eg, pregnant women, young children, or people over 65 years old) were more likely to feel overwhelmed by COVID-19 information. However, inconsistent with previous studies showing that people who perceived themselves to have less-than-excellent health were more susceptible to information overload [77], our results indicated an insignificant relationship between chronic illness records and perceived information overload about COVID-19. More studies are needed to explore the association between pre-existing health conditions and perceived information overload during the COVID-19 pandemic.

As for the effect of information-seeking behavior on perceived information overload (second research question), we found that the use of traditional media (eg, newspaper, television) for seeking COVID-19 information was positively associated with perceived information overload. In contrast, the effect of getting COVID-19 information from online news outlets was not significant. Given that the internet and online media contribute to the over-proliferation of health information available to the lay public [51], such results seem unreasonable. A possible explanation is that, while offline seekers have little or no control over what is aired but receive information passively, online information seekers can adjust the search terms anytime to suit their search needs, thereby getting tailored information. Compared with traditional media, internet media are active channels that require greater cognitive effort and are often used by highly motivated and engaged individuals [78]. Moreover, online users can easily search other websites for clarification or fact-checking whenever they find the information received from a single online source is unreliable [17]. Thus, the motivation to authenticate earlier information obtained may prohibit online seekers from being overwhelmed with the information received. Future studies should take the route of information acquisition (eg, active search vs passive exposure) into account.

Our results underscore the critical role of trust in information sources in crisis management. Interestingly, the perceived reliability of COVID-19 information from various media channels exerted differential effects on perceived information overload in our study. Perceived trustworthiness of COVID-19 information from television was negatively associated with perceived information overload. It was consistent with prior research that higher trust in information sources predicted less distress by information and benefited crisis management [79]. However, respondents were more likely to experience information overload when they perceived COVID-19 information from online media (ie, online news outlets, social media) as more trustworthy. It may be because misinformation, disinformation, and rumors about COVID-19 were more prevalent on online media than other forms of media [44], which may engender greater confusion and require more cognitive effort to process such inaccurate and inconsistent information for those who believe in the information. As mentioned earlier, the frequency of seeking COVID-19 information online was not associated with perceived information overload. Thus, the level of perceived information overload seems more attributable to the perceived credibility of information on the internet rather than the online information-seeking behavior.

As for the third research question about the psychological and behavioral consequences of information overload, we found that an overabundance of COVID-19 information can harm mental well-being by increasing the likelihood of experiencing posttraumatic stress disorder. Such a finding was consistent with previous studies about the negative psychological impact of information overload [17]. During an unprecedented global pandemic like COVID-19, people are understandably attentive to health information and, by doing so, try to reduce the negative affect caused by the previously unknown and unpredictable disease. However, when information flow exceeds one’s capacity to process, the perceived information overload may cause a detrimental effect on psychological well-being. Moreover, deteriorated mental health caused by information overload may lead to information avoidance when people deliberately avoid seeking health information to lessen the cognitive burden and negative affect [76]. Information avoidance is detrimental for public health because the acquisition of health information helps individuals make informed medical decisions and engage in preventive behaviors [80]. Given the unintended consequences of information overload, our study highlighted the importance of keeping a balance between information transparency and information overload.

However, our results did not show a significant effect of perceived information overload on protective behaviors during the COVID-19 pandemic, which was consistent with a study conducted during the COVID-19 pandemic in Korea [25]. Despite no direct effect, information overload may indirectly reduce compliance via increasing heuristic processing and decreasing systematic processing [25]. Compared with systematic processing of information, judgments based on heuristic processing tend to be less stable and weakly tied to subsequent behaviors [81], which may discourage one’s willingness to adopt protective measures. When the risk perception of COVID-19 decreases and the anxiety associated with this pandemic fade over time, the detrimental effect of perceived information overload on protective behaviors may emerge. Future studies are warranted to investigate the behavioral consequences of information overload in different time frames.

The fourth research question focused on who was more vulnerable to the psychological and behavioral consequences of information overload. Since there was no significant effect of perceived information overload on protective behaviors, we did not further examine whether such an effect may be different across various sociodemographic characteristics. As for psychological distress, we found that women and people with a higher SES were more vulnerable to the adverse effects of perceived information overload on psychological well-being. Female respondents were more likely to experience psychological distress when exposed to information overload, possibly because women are more likely than men to perceive the pandemic as a severe health problem and to agree and comply with restraining measures [53]. Besides, we speculated that people with a higher SES might be less psychologically resilient in the face of the COVID-19 pandemic and thus perceived more significant information overload. Previous studies have suggested that people with a higher SES were more concerned and worried about the pandemic [82] and may have been experiencing a more drastic relative change in their lifestyles, leading to a sharper decline in their psychological well-being.

Meanwhile, individuals with a lower SES tend to experience more everyday stressors even when there is no global pandemic. Thus, they may not undergo a significant change in their subjective well-being when reading pessimistic news about COVID-19. Besides, people with higher education care more about the quality of information and may feel frustrated if they cannot find trustworthy sources during the pandemic. Given the ambiguous information and inconsistent guidance about COVID-19, especially during the early stage of the outbreak, it is hard to identify valuable and reliable information even for people with a higher SES who are believed to have more access to health information and a higher ability to process such information.

Since information overload can harm mental well-being and potentially reduce compliance during the pandemic, it is urgent to manage the overload of information that exceeds people’s cognitive ability to process. From the information provider side, government and media should disseminate evidence-based and transparent information swiftly and widely among the public. Social media technology companies must constantly review content shared on their platforms and closely verify the reliability of information related to the pandemic. Since there are stratified mechanisms and consequences of information overload as shown in this study, information policies and management should be accordingly “stratified” as well. It is essential to develop efficient health communication strategies targeting people with different sociodemographic characteristics. Certain subgroups may be more frustrated with the uncertainty caused by the pandemic and eagerly look for sources to fill their information needs. It is necessary to formulate different information dissemination strategies in terms of information content and channels for different groups. It is also essential to establish interventions to help people vulnerable to information overload to cope with information anxiety and mental health concerns.

On the receiver side, individuals should carry out an “information diet” by controlling the extent and type of information they consume. Researchers have suggested that people visit authentic and official websites for COVID-19 information and try to verify suspicious news on one of the many fact-checking websites dedicated to debunking myths [8,83]. For example, the World Health Organization’s (WHO) risk communication team launched a new information platform known as the WHO Information Network for Epidemics, which actively reports details related to COVID-19. A team of WHO “myth busters” works with search and media companies like Facebook, Google, Pinterest, Tencent, Twitter, TikTok, YouTube, and others to counter the spread of rumors. Also, enhancing information literacy skills, particularly health literacy skills, can facilitate the critical evaluation of health information and help people make appropriate health-related decisions. It is argued that information and health literacy is vital to reducing information overload and its consequences [84,85]. Thus, extensive training and guidelines for improving information and health literacy should be promoted to the public, particularly regarding crisis management.

### Limitations

Despite the significant findings, this study is not without limitations. First, the data were obtained from a cross-sectional survey, and it is hard to ascertain the causal relationship between variables. For example, our results showed a negative effect of perceived information overload on psychological well-being. However, trait anxiety was significantly associated with higher levels of perceived information overload [24]. Thus, longitudinal studies are needed to ascertain the psychological consequences of information overload. Second, only one item was used to measure perceived information overload, which presents a significant limitation of this study. We recognized that information overload is a multidimensional construct and a single proxy item limits our ability to make clear-cut generalizations from our findings. Future studies may adopt validated scales regarding COVID-19 information overload (eg, Corona Information Overload Scale [65]). Third, this study has limited information on knowledge and attitudes related to COVID-19, though we have adjusted for perceived susceptibility and perceived severity of COVID-19 in the analysis. Future studies may further examine how knowledge and attitudes may be associated with information overload.

### Conclusion

Perceptions of information overload are prevalent during the COVID-19 pandemic and have caused significant psychological and behavioral consequences. This study is among the first to examine how the antecedents and consequences of perceived information overload vary between different sociodemographic groups among the Asian population. A cross-sectional survey with representative data of 10,063 residents in 6 jurisdictions in Asia was conducted in May 2020. Regression results confirmed a positive relationship between perceived information overload and psychological distress. Also, people with a higher SES were more exposed to information overload about COVID-19 and suffered more psychological distress because of perceived information overload. Our findings suggested that the provision of trustworthy information and reduction of the perceived information overload can significantly ameliorate psychological distress during the pandemic. Effective policies and interventions should be promoted to target higher-SES populations who are more susceptible to the occurrence and adverse psychological influence of perceived information overload.

## Appendix

Multimedia Appendix 1 Tests of Ordinal Least Squares (OLS) assumptions.

## Abbreviations

CHERRIES: Checklist for Reporting Results of Internet E-Surveys
DSM-5: Diagnostic and Statistical Manual of Mental Disorders 5
MERS: Middle East Respiratory Syndrome
OLS: ordinary least squares
PCL-5: Posttraumatic Stress Disorder Checklist for DSM-5
SARS: Severe Acute Respiratory Syndrome
SES: socioeconomic status
WHO: World Health Organization

## References

[1] Mandhana N (2021). Asia Suffers Outbreaks Where Covid-19 Had Seemed Beaten. Wall Street Journal.

[2] Coronavirus updates. Worldometer.

[3] Jin L, Chen X, Lin F, Zou Y, Gao H (2022). Does education matter for psychological recovery amidst the COVID-19 pandemic? Evidence from a panel survey in Hubei, China. Anxiety Stress Coping.

[4] Tso IF, Park S (2020). Alarming levels of psychiatric symptoms and the role of loneliness during the COVID-19 epidemic: A case study of Hong Kong. Psychiatry Res.

[5] Lee H, Dean D, Baxter T, Griffith T, Park S (2021). Deterioration of mental health despite successful control of the COVID-19 pandemic in South Korea. Psychiatry Res.

[6] Li D, Ko N, Chen Y, Wang P, Chang Y, Yen C, Lu W (2020). COVID-19-related factors associated with sleep disturbance and suicidal thoughts among the Taiwanese public: a Facebook survey. Int J Environ Res Public Health.

[7] (2020). Novel coronavirus (2019-nCov): situation report-13. World Health Organization.

[8] Rathore F, Farooq F (2020). Information overload and infodemic in the COVID-19 pandemic. J Pak Med Assoc.

[9] Taha S, Matheson K, Cronin T, Anisman H (2014). Intolerance of uncertainty, appraisals, coping, and anxiety: the case of the 2009 H1N1 pandemic. Br J Health Psychol.

[10] Fischhoff B, Wong-Parodi G, Garfin DR, Holman EA, Silver RC (2018). Public understanding of Ebola risks: mastering an unfamiliar threat. Risk Anal.

[11] Nagler RH, Vogel RI, Gollust SE, Rothman AJ, Fowler EF, Yzer MC (2020). Public perceptions of conflicting information surrounding COVID-19: Results from a nationally representative survey of U.S. adults. PLoS One.

[12] Garfin DR, Silver RC, Holman EA (2020). The novel coronavirus (COVID-2019) outbreak: Amplification of public health consequences by media exposure. Health Psychol.

[13] Sweeny K, Melnyk D, Miller W, Shepperd JA (2010). Information avoidance: who, what, when, and why. Review of General Psychology.

[14] Jensen JD, Carcioppolo N, King AJ, Scherr CL, Jones CL, Niederdieppe J (2014). The cancer information overload (CIO) scale: establishing predictive and discriminant validity. Patient Educ Couns.

[15] Niederdeppe J, Fowler E, Goldstein K, Pribble J (2010). Does local television news coverage cultivate fatalistic beliefs about cancer prevention?. J Commun.

[16] Liu C, Kuo K (2016). Does information overload prevent chronic patients from reading self-management educational materials?. Int J Med Inform.

[17] Swar B, Hameed T, Reychav I (2017). Information overload, psychological ill-being, and behavioral intention to continue online healthcare information search. Computers in Human Behavior.

[18] Crook B, Stephens KK, Pastorek AE, Mackert M, Donovan EE (2016). Sharing health information and influencing behavioral intentions: the role of health literacy, information overload, and the internet in the diffusion of healthy heart information. Health Commun.

[19] Niederdeppe J, Lee T, Robbins R, Kim HK, Kresovich A, Kirshenblat D, Standridge K, Clarke CE, Jensen J, Fowler EF (2014). Content and effects of news stories about uncertain cancer causes and preventive behaviors. Health Commun.

[20] No authors listed (2020). The COVID-19 infodemic. The Lancet Infectious Diseases.

[21] Mohammed M, Sha'aban A, Jatau AI, Yunusa I, Isa AM, Wada AS, Obamiro K, Zainal H, Ibrahim B (2022). Assessment of COVID-19 information overload among the general public. J Racial Ethn Health Disparities.

[22] Liu H, Liu W, Yoganathan V, Osburg V (2021). COVID-19 information overload and generation Z's social media discontinuance intention during the pandemic lockdown. Technol Forecast Soc Change.

[23] Bala R, Srivastava A, Ningthoujam GD, Potsangbam T, Oinam A, Anal CL (2021). An observational study in Manipur State, India on preventive behavior influenced by social media during the COVID-19 pandemic mediated by cyberchondria and information overload. J Prev Med Public Health.

[24] Fan J, Smith AP (2021). Information overload, wellbeing and COVID-19: a survey in China. Behav Sci (Basel).

[25] Hong H, Kim HJ (2020). Antecedents and consequences of information overload in the COVID-19 pandemic. Int J Environ Res Public Health.

[26] Sokolov M (2020). The pandemic infodemic: how social media helps (and hurts) during the coronavirus outbreak. The Drum.

[27] Yang Y, Liu K, Li S, Shu M (2020). Social media activities, emotion regulation strategies, and their interactions on people's mental health in COVID-19 pandemic. Int J Environ Res Public Health.

[28] Misra S, Stokols D (2011). Psychological and health outcomes of perceived information overload. Environment and Behavior.

[29] Khaleel I, Wimmer BC, Peterson GM, Zaidi STR, Roehrer E, Cummings E, Lee K (2020). Health information overload among health consumers: A scoping review. Patient Educ Couns.

[30] Bawden D, Robinson L (2008). The dark side of information: overload, anxiety and other paradoxes and pathologies. Journal of Information Science.

[31] Chan YM, Huang H (2013). Weight management information overload challenges in 2007 HINTS: socioeconomic, health status and behaviors correlates. Journal of Consumer Health On the Internet.

[32] Akin LK (1997). Information overload: A multi-disciplinary explication and citation ranking within three selected disciplines: Library studies, psychology/psychiatry, and consumer science: 1960-1996. Texas Woman's University.

[33] Ji Q, Ha L, Sypher U (2014). The role of news media use and demographic characteristics in the possibility of information overload prediction. International Journal of Communication.

[34] Lagoe C, Atkin D (2015). Health anxiety in the digital age: An exploration of psychological determinants of online health information seeking. Computers in Human Behavior.

[35] York C (2013). Overloaded by the news: effects of news exposure and enjoyment on reporting information overload. Communication Research Reports.

[36] Chae J, Lee C, Jensen JD (2016). Correlates of cancer information overload: focusing on individual ability and motivation. Health Commun.

[37] Obamiro K, Lee K (2019). Information overload in patients with atrial fibrillation: Can the cancer information overload (CIO) scale be used?. Patient Educ Couns.

[38] Kim K, Lustria ML, Burke D, Kwon N (2007). Predictors of cancer information overload: findings from a national survey. Information Research.

[39] Vollmer Dahlke D, Fair K, Hong YA, Beaudoin CE, Pulczinski J, Ory MG (2015). Apps seeking theories: results of a study on the use of health behavior change theories in cancer survivorship mobile apps. JMIR Mhealth Uhealth.

[40] Jensen JD, Liu M, Carcioppolo N, John KK, Krakow M, Sun Y (2017). Health information seeking and scanning among US adults aged 50-75 years: Testing a key postulate of the information overload model. Health Informatics J.

[41] Gurmankin AD, Viswanath V The impact of health information overload on attention to information and cancer risk reduction behavior.

[42] Chao M, Xue D, Liu T, Yang H, Hall BJ (2020). Media use and acute psychological outcomes during COVID-19 outbreak in China. J Anxiety Disord.

[43] Merchant RM, Lurie N (2020). Social media and emergency preparedness in response to novel coronavirus. JAMA.

[44] Bastani P, Bahrami M (2020). COVID-19 related misinformation on social media: a qualitative study from Iran. J Med Internet Res.

[45] Eppler MJ, Mengis J (2004). Side-effects of the e-society: The causes of information overload and possible countermeasures.

[46] Holmes BJ (2008). Communicating about emerging infectious disease: The importance of research. Health, Risk & Society.

[47] Tausczik Y, Faasse K, Pennebaker JW, Petrie KJ (2012). Public anxiety and information seeking following the H1N1 outbreak: blogs, newspaper articles, and Wikipedia visits. Health Commun.

[48] Chaiken S, Ledgerwood A, Van Lange PAM, Kruglanski AW, Higgins ET (2012). A theory of heuristic and systematic information processing. Handbook of theories of social psychology.

[49] Wheaton MG, Abramowitz JS, Berman NC, Fabricant LE, Olatunji BO (2011). Psychological predictors of anxiety in response to the H1N1 (Swine Flu) pandemic. Cogn Ther Res.

[50] Thompson RR, Garfin DR, Holman EA, Silver RC (2017). Distress, worry, and functioning following a global health crisis: a national study of Americans’ responses to Ebola. Clinical Psychological Science.

[51] Wang C, Pan R, Wan X, Tan Y, Xu L, Ho CS, Ho RC (2020). Immediate psychological responses and associated factors during the initial stage of the 2019 Coronavirus disease (COVID-19) epidemic among the general population in China. Int J Environ Res Public Health.

[52] Gao J, Zheng P, Jia Y, Chen H, Mao Y, Chen S, Wang Y, Fu H, Dai J (2020). Mental health problems and social media exposure during COVID-19 outbreak. PLoS One.

[53] Ko N, Lu W, Chen Y, Li D, Wang P, Hsu S, Chen C, Lin Y, Chang Y, Yen C (2020). COVID-19-related information sources and psychological well-being: An online survey study in Taiwan. Brain Behav Immun.

[54] Viswanath K, Nagler RH, Bigman-Galimore CA, McCauley MP, Jung M, Ramanadhan S (2012). The communications revolution and health inequalities in the 21st Century: implications for cancer control. Cancer Epidemiol Biomarkers Prev.

[55] Molina Y, Zimmermann K, Carnahan LR, Paulsey E, Bigman CA, Khare MM, Zahnd W, Jenkins WD (2018). Rural women's perceptions about cancer disparities and contributing factors: a call to communication. J Cancer Educ.

[56] Jensen JD, King AJ, Carcioppolo N, Krakow M, Samadder NJ, Morgan S (2014). Comparing tailored and narrative worksite interventions at increasing colonoscopy adherence in adults 50-75: a randomized controlled trial. Soc Sci Med.

[57] Han PKJ, Moser RP, Klein WMP (2007). Perceived ambiguity about cancer prevention recommendations: associations with cancer-related perceptions and behaviours in a US population survey. Health Expect.

[58] Niederdeppe J, Levy AG (2007). Fatalistic beliefs about cancer prevention and three prevention behaviors. Cancer Epidemiology Biomarkers & Prevention.

[59] Farooq A, Laato S, Islam AKMN (2020). Impact of online information on self-isolation intention during the COVID-19 pandemic: cross-sectional study. J Med Internet Res.

[60] Siebenhaar KU, Köther AK, Alpers GW (2020). Dealing with the COVID-19 infodemic: distress by information, information avoidance, and compliance with preventive measures. Front Psychol.

[61] Pickles K, Cvejic E, Nickel B, Copp T, Bonner C, Leask J, Ayre J, Batcup C, Cornell S, Dakin T, Dodd RH, Isautier JMJ, McCaffery KJ (2021). COVID-19 misinformation trends in Australia: prospective longitudinal national survey. J Med Internet Res.

[62] Voigt T, Holtz V, Niemiec E, Howard H, Middleton A, Prainsack B (2020). Willingness to donate genomic and other medical data: results from Germany. Eur J Hum Genet.

[63] Baker R, Blumberg SJ, Brick JM, Couper MP, Courtright M, Dennis JM, Dillman D, Frankel MR, Garland P, Groves RM, Kennedy C, Krosnick J, Lavrakas PJ, Lee S, Link M, Piekarski L, Rao K, Thomas RK, Zahs D (2010). Research Synthesis: AAPOR Report on Online Panels. Public Opinion Quarterly.

[64] Blom AG, Gathmann C, Krieger U (2015). Setting up an online panel representative of the general population. Field Methods.

[65] Stanley M, Roycroft J, Amaya A, Dever JA, Srivastav A (2020). The effectiveness of incentives on completion rates, data quality, and nonresponse bias in a probability-based internet panel survey. Field Methods.

[66] Eysenbach G (2004). Improving the quality of Web surveys: the Checklist for Reporting Results of Internet E-Surveys (CHERRIES). J Med Internet Res.

[67] Lee S, Yuen S, Or N, Cheng EW, Yue R (2022). Pandemic vulnerability, policy feedback and support for immigration: Evidence from Asia. Br J Soc Psychol.

[68] Weathers F, Litz B, Keane T, Palmieri P, Marx B, Schnurr P (2013). PTSD Checklist for DSM-5 (PCL-5). U.S. Department of Veterans Affairs.

[69] Sarkhel S, Bakhla A, Praharaj S, Ghosal M (2020). Information overload regarding COVID-19: Adaptation and validation of the cancer information overload scale. Indian J Psychiatry.

[70] Schmidt A, Finan C (2018). Linear regression and the normality assumption. J Clin Epidemiol.

[71] Hirschler B (2021). Information overload. Brunswick Group.

[72] Bonoil M, McIsaac M, Xu L, Wuliji T, Diallo K, Campbell J (2019). Gender equity in the health workforce: analysis of 104 countries. World Health Organization.

[73] Galasso V, Pons V, Profeta P, Becher M, Brouard S, Foucault M (2020). Gender differences in COVID-19 attitudes and behavior: Panel evidence from eight countries. Proc Natl Acad Sci U S A.

[74] Zhao X, Fan J, Basnyat I, Hu B (2020). Online health information seeking using "#COVID-19 Patient Seeking Help" on Weibo in Wuhan, China: descriptive study. J Med Internet Res.

[75] Lee K, Hoti K, Hughes JD, Emmerton LM (2015). Consumer Use of "Dr Google": a survey on health information-seeking behaviors and navigational needs. J Med Internet Res.

[76] Zickuhr K (2010). Generations 2010. Pew Internet Research.

[77] Fox S, Rainie L (2002). E-patients and the online health care revolution. Physician Exec.

[78] Chae J, Lee C, Kim K (2020). Prevalence, predictors, and psychosocial mechanism of cancer information avoidance: findings from a national survey of U.S. adults. Health Commun.

[79] Rubin GJ, Amlôt R, Page L, Wessely S (2009). Public perceptions, anxiety, and behaviour change in relation to the swine flu outbreak: cross sectional telephone survey. BMJ.

[80] Soroya SH, Farooq A, Mahmood K, Isoaho J, Zara S (2021). From information seeking to information avoidance: Understanding the health information behavior during a global health crisis. Inf Process Manag.

[81] Trumbo CW (2002). Information processing and risk perception: An adaptation of the heuristic-systematic model. Journal of Communication.

[82] Talev M (2020). Axios-Ipsos coronavirus index: Rich sheltered, poor shafted amid virus. Axios.

[83] Chen X, Gao H, Zou Y, Lin F (2020). Changes in psychological wellbeing, attitude and information-seeking behaviour among people at the epicentre of the COVID-19 pandemic: a panel survey of residents in Hubei province, China. Epidemiol Infect.

[84] Klerings I, Weinhandl AS, Thaler KJ (2015). Information overload in healthcare: too much of a good thing?. Z Evid Fortbild Qual Gesundhwes.

[85] Lee T, Lee B, Lee-Geiller S (2020). The effects of information literacy on trust in government websites: Evidence from an online experiment. International Journal of Information Management.

