# Supplementary material for: Stratified Impacts of the Infodemic During the COVID-19 Pandemic: Cross-sectional Survey in 6 Asian Jurisdictions
Source: J Med Internet Res. 2022 Mar 22;24(3):e31088. doi: 10.2196/31088 (PMC8942092; doi:10.2196/31088)
Supplement: Multimedia Appendix 1 [file jmir_v24i3e31088_app1.docx]

Appendix 1.

Tests of Ordinal Least Squares (OLS) assumptions.

Some important assumptions of OLS regression include 1) linearity, 2) homoscedasticity, 3) normality of errors, 4) no perfect multicollinearity, and 5) independence/no autocorrelation (Schmidt & Finan, 2018). As the dependence/ autocorrelation issue usually occurs in multilevel or time-series data, there should be a problem for our data. We have used graphic or numerical tests to verify the other OLS assumptions (1-4) for all of the three dependent variables: 1) perceived information overload, 2) psychological distress, and 3) preventive behavior.

Regression of perceived information overload

1) For testing the assumption of linearity, we used the added-variable plot, which is a partial-regression leverage plot to identify any potential non-linear relationship and outliers. The results show limited concern about this issue.


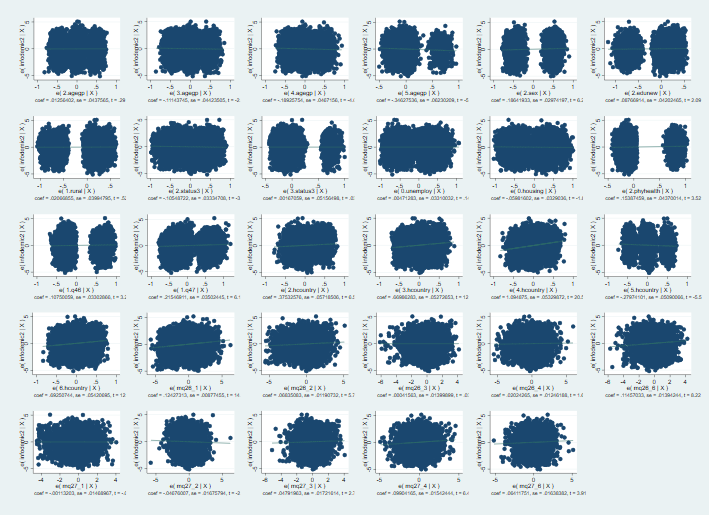


2) For checking the normality of residuals, we plotted the distribution of the residuals. The plot seems to show only a minor deviation from the normality. We can accept that the residuals are close to a normal distribution.

3) For checking homoscedasticity of residuals, we used Breusch-Pagan / Cook-Weisberg test for heteroskedasticity. The results rejected the null hypothesis of homoscedasticity and showed a certain level of heteroskedasticity. a relatively straightforward solution is to replace the standard errors with larger heteroscedastic robust standard errors (Schmidt & Finan, 2018). We thus used OLS regression with robust standard error. We also used Weighted Least Squares regression, and the results were similar. For the sake of space, we only reported results from OLS regression with robust standard errors.

4) The VIF is 1.68, which represents no perfect multicollinearity.

Regression of psychological distress

1) We used the added-variable plots to test the assumption of linearity, which showed limited concern for this issue.


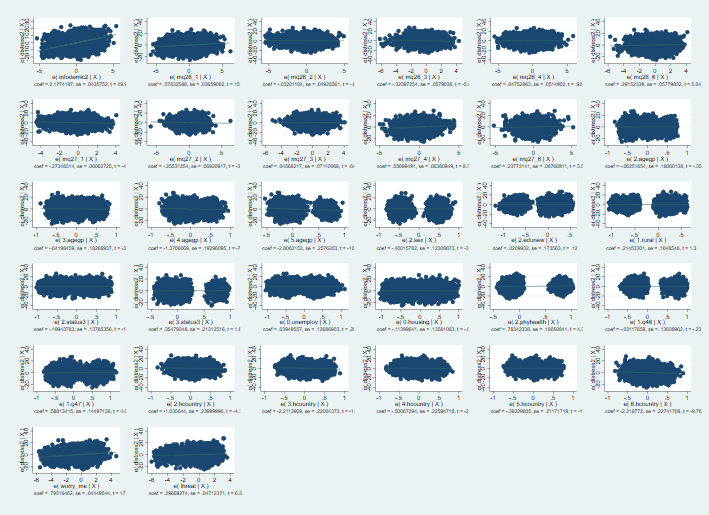


2) For checking the normality of residuals, we graphed a histogram plot of the distribution of the residuals. The graph showed that the residuals are close to a normal distribution.

3) For checking the homoscedasticity of residuals, we used Breusch-Pagan / Cook-Weisberg test for heteroskedasticity. The results cannot reject the null hypothesis of homoscedasticity and confirmed the homoscedasticity of residuals,

4) The VIF is 1.66, which represents no perfect multicollinearity.

Regression of preventive behavior

1) The added-variable plots showed some deviation of linearity.


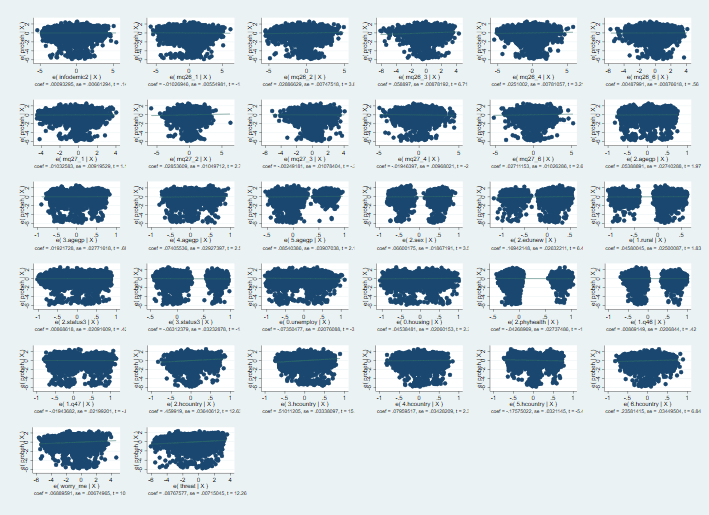


2) The plot of the distribution of the residuals showed that the residuals are close to a normal distribution.

3) For checking homoscedasticity of residuals, we used Breusch-Pagan / Cook-Weisberg test for heteroskedasticity. The results rejected the null hyothesis of homoscedasticity and showed certain level of heteroskedasticity. We used OLS regression with robust standard error to fix this issue. We also used Weighted Least Squares regression and the results were similar. For the sake of space, we only reported results from OLS regression with robust standard errors.

4) The VIF is 1.66, which represents no perfect multicollinearity.

Reference:

Schmidt, A. F., & Finan, C. (2018). Linear regression and the normality assumption. Journal of clinical epidemiology, 98, 146-151.
